# Single-center observational analysis of a pharmacist-driven antibiotic time-out targeting antipseudomonal drugs

**DOI:** 10.1017/ash.2025.6

**Published:** 2025-02-17

**Authors:** Carina Diaz, Alice Margulis Landayan, Lorenzo Porras, Sonia Samsundar, Jorge Murillo, Timothy P. Gauthier

**Affiliations:** 1 Department of Pharmacy, South Miami Hospital, South Miami, FL, USA; 2 Department of Medicine, South Miami Hospital, South Miami, FL, USA; 3 Clinical Pharmacy Enterprise, Baptist Health South Florida, Miami, FL, USA

## Abstract

This study found that implementation of a pharmacist-driven antipseudomonal antibiotic time-out at a 450-bed community hospital led to a 5% reduction in the use of targeted antipseudomonal antibiotics (*P* = .12), which may be clinically meaningful as it extrapolates to approximately 1,800 avoided days of therapy annually.

*Pseudomonas aeruginosa* is a non-fermenting gram-negative bacillus and important cause of nosocomial infections.^1^ The misuse of antimicrobial agents targeting *P. aeruginosa* can negatively contribute to the risk of adverse drug reactions and ecological consequences of antibiotic use.^2^ The Centers for Disease Control and Prevention (CDC) Core Elements of Hospital Antibiotic Stewardship Programs emphasize the importance of implementing actions such as prospective audit and feedback and antimicrobial “time-outs” as antimicrobial stewardship measures to improve antibiotic use.^3^ Pharmacist expertise is one of the CDC Core Elements, which highlights pharmacists as key team members in promoting optimal antimicrobial stewardship practices. Antimicrobial stewardship programs (ASP) have demonstrated positive impacts on antipseudomonal use.^4^ This study aimed to assess the impact of a pharmacist-driven antipseudomonal antibiotic time-out as a mechanism for reducing unnecessary antipseudomonal antibiotic use.

## Methods

This was a retrospective evaluation of a pharmacist-driven antipseudomonal antibiotic time-out at a 453-bed community hospital comparing January 1, 2023 to February 28, 2023 (pre-period) versus December 12, 2023 to February 29, 2024 (post-period). During weekdays, a report utilizing an electronic reporting system, Cerner Discern Analytics 2.0^TM^, for target antipseudomonal (piperacillin/tazobactam, cefepime, aztreonam, ciprofloxacin, and levofloxacin) orders was obtained. Established pharmacists (an infectious diseases [ID] pharmacist, a critical care pharmacist, or a pharmacist resident) were assigned to identify interventions. Some interventions were completed using long-standing hospital protocols without the need to contact providers (eg, renal dosing), while non-protocolized interventions required provider contact and approval (eg, drug selection).

The primary endpoint was the rate of antipseudomonal antibiotic use, assessed by measuring the proportion of target antipseudomonal antibiotics out of all other systemic antibacterial days of therapy (DOT) in the pre- and post-periods, adjusted to days present to account for census variation.^5,6^ Data for this endpoint were extracted from the VigiLanz® clinical surveillance platform. Primary endpoint data were used to estimate an annualized impact of the intervention. Hospital-wide total DOT for target antipseudomonal agents was secondarily analyzed as part of this quantitative assessment.

A qualitative component of the study was undertaken to evaluate the intervention arm, which included patients 18 years of age or older who received at least 1 dose of a target antipseudomonal agent while hospitalized. Patients were excluded if pregnant, incarcerated, taking chronic suppressive or prophylactic antimicrobial therapy, or receiving comfort measures only or hospice care. Endpoints included pharmacist antibiotic intervention details, intervention acceptance rate, antipseudomonal agent duration of therapy, incidence of *C*. *difficile* infection (CDI), and length of hospital stay.

For statistical analysis, categorical data were analyzed using the chi-square test. Statistical significance was determined using *P* < .05. Additionally, descriptive statistics including medians and interquartile ranges for continuous variables and percentages for categorical variables were used. This study was deemed exempt by the Institutional Review Board.

## Results

**Figure 1.**
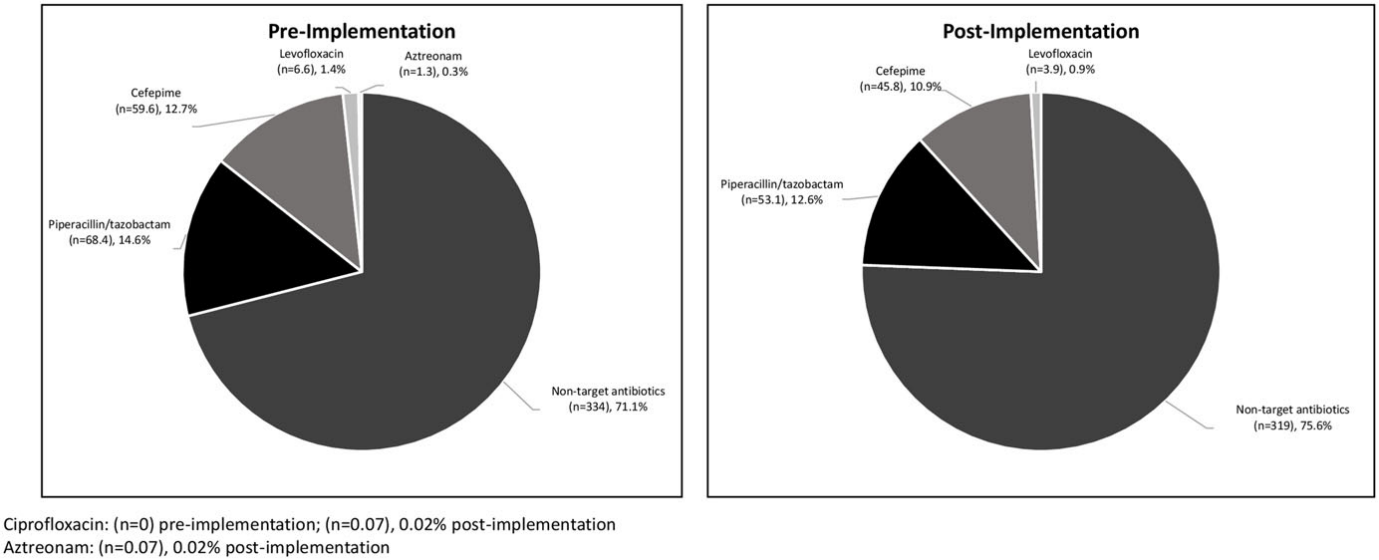
Total days of therapy per 1,000 days present.

The proportion of target antipseudomonal versus all other systemic antibiotic DOT adjusted to days present was 136 of 470 (29%) for the pre-implementation group compared to 103 of 423 (24%) for the post-implementation group (*P* = .12). Approximating an average 3,000 monthly antimicrobial DOT, census of 6,500 days present, and baseline target DOT of 870, a 5% reduction in target DOTs is expected to produce 150 less target DOTs per month. This extrapolates to an estimated reduction of 1,800 target DOTs per year. The total DOT per 1,000 days present per antipseudomonal agent is shown in Figure 1. Days present were 12,816 for the pre-implementation group and 14,965 for the post-implementation group.

For the qualitative post-implementation component of the analysis, 537 patients were reviewed, with 200 patients (37%) intervened on with a total of 255 interventions. Baseline characteristics for this population are displayed in Table 1. The interventions completed were antimicrobial de-escalation (n = 76), order stop date modification (n = 63), antimicrobial discontinuation (n = 36), allergy documentation updated (n = 31), antimicrobial escalation (n = 13), antimicrobial dose adjustment (n = 11), ID provider consulted (n = 9), and other interventions (n = 16). The pharmacist intervention acceptance rate was 98.8% (255 accepted of 258 recommended). A total of 45 interventions (17.6%) were completed per institutional protocols. Antimicrobial de-escalations were completed primarily in patients with intra-abdominal infection (n = 36, 47%) and genitourinary tract infection (n = 20, 26%). Antibiotic de-escalations were performed for piperacillin/tazobactam (n = 45, 59%), cefepime (n = 24, 32%), and levofloxacin (n = 7, 9%). Antimicrobial discontinuations were completed most commonly in patients with pulmonary infection (n = 10, 28%) and skin and soft tissue infection (n = 8, 22%).

**Table 1.** Baseline characteristics for qualitative analysis

Characteristic	Post-implementation (n = 200)
Median age (IQR)-years	72.5 (63–83)
Male gender, n (%)	111 (55.5)
Median weight (IQR)-kg	74.8 (63–90)
Antimicrobial allergy, n (%)^1^ Penicillin Cephalosporin Sulfa Other	55 (27.5) 33 (16.5) 4 (2) 12 (6) 16 (8)
Admission to intensive care unit, n (%)	79 (39.5)
Infection type, n (%)^2^ Bacteremia Pulmonary infection * Community-acquired* * Hospital-acquired* * Aspiration* * Ventilator-acquired* * Other* Intra-abdominal infection * Appendicitis* * Cholecystitis* * Diverticulitis* * Other* Genitourinary tract infection * Cystitis* * Pyelonephritis* * Other* Skin and soft tissue infection * Non-purulent* * Abscess/purulent* * Other* Other infection types	18 (9) 78 (39) 38 (19) 21 (10.5) 13 (6.5) 4 (2) 2 (1) 51 (25.5) 9 (4.5) 7 (3.5) 7 (3.5) 28 (14) 31 (15.5) 27 (13.5) 3 (1.5) 1 (0.5) 24 (12) 7 (3.5) 2 (1) 15 (7.5) 18 (9)

1 Patients may have an allergy to more than one antibiotic.

2 Patients may have more than one type of infection.

In the qualitative analysis, the median duration of therapy was 70 hours for cefepime, 46 hours for piperacillin/tazobactam, 27 hours for levofloxacin, 23 hours for aztreonam, and 0 hours for ciprofloxacin. One patient developed CDI after exposure to piperacillin/tazobactam. The median length of hospital stay was 7 days.

## Discussion

Implementation of a pharmacist-driven antipseudomonal antibiotic time-out within a community hospital was not found to produce a statistically significant difference in antipseudomonal consumption; however, the reduction achieved was considered to be clinically meaningful. A potential decrease of 1,800 DOTs annually translates to less patient exposure/risk, less pressure to induce resistance, and reduced healthcare costs. Additionally, these findings align with CDC recommendations to implement antibiotic time-outs, with these data supporting such activities specifically targeting antipseudomonal agents.

Cefepime and piperacillin/tazobactam were the agents with the highest utilization in this study. These drugs are important targets due to their broad spectrum of activity and high rate of use in hospitals nationwide. Providing context to how reducing exposure mitigates risk, the risk of multidrug resistance emergence has been found to increase by 8% for each day of additional exposure to these agents.^7^ Antimicrobial time-outs ensure appropriate use of these medications by facilitating higher rates of antibiotic de-escalation when appropriate. For instance, the frequency of de-escalation in this study highlights antimicrobial de-escalation as a key intervention for these medications and supports the need for continuous implementation of antipseudomonal time-outs at the study institution.

Antimicrobial audit and feedback ensure systematic assessment of broad-spectrum antibiotics and allow for optimal use of antibiotics and duration of therapy. During the time-out implementation, pharmacists were able to intervene in patients hospitalized in different units, demonstrating that pharmacists can contribute to improved use of antipseudomonal agents in various hospitalized patient populations. In this study, the acceptance rate of 98.8% exceeded that in previous literature.^8^ The extremely high acceptance rate of pharmacist-recommended interventions seen during the implementation period may be due to the well-established ASP and the strong working relationship between pharmacists and providers.

Limitations of this study include a single-center design, which can potentially limit the generalizability of results. However, the results can be relevant to other medium-sized hospitals by supporting the development of similar antimicrobial services. Qualitative secondary outcomes were not assessed for the pre-implementation group as the collection of information on the patients that were intervened on was more impactful to describe the process to achieve change, and notably no major quality-related concerns were identified.

This project led to several new initiatives within the hospital, including the development of education for providers and pharmacists focused on indications for antipseudomonal therapy and the implementation of additional rules for the automated clinical decision support system.

Implementation of this pharmacist-driven antipseudomonal antibiotic time-out at a 450-bed community hospital led to a meaningful reduction in the use of targeted antipseudomonal antibiotics, which extrapolates to approximately 1,800 avoided days of therapy annually. Facilities that have substantial piperacillin-tazobactam or cefepime utilization may have the most potential for benefit from adopting this targeted antibiotic time-out intervention.
